# Interbirth interval and maternal anaemia in 21 sub-Saharan African countries: A fractional-polynomial analysis

**DOI:** 10.1371/journal.pone.0275155

**Published:** 2022-09-23

**Authors:** Kalayu Brhane Mruts, Amanuel Tesfay Gebremedhin, Gizachew A. Tessema, Jane A. Scott, Gavin Pereira

**Affiliations:** 1 School of Population Health, Curtin University, Perth, Australia; 2 School of Public Health, University of Adelaide, Adelaide, Australia; 3 Centre for Fertility and Health (CeFH), Norwegian Institute of Public Health, Oslo, Norway; 4 enAble Institute, Curtin University, Perth, Australia; FHI360, UNITED STATES

## Abstract

**Background:**

Maternal anaemia is a global public health problem contributing to adverse maternal and perinatal outcomes. In addition to other risk factors, interbirth interval has been identified as a potentially modifiable risk factor of maternal anaemia. However, the current evidence for the association between interbirth interval and maternal anaemia remains inconclusive. Hence, this study examined the association between the interbirth interval and maternal anaemia in sub-Saharan Africa.

**Methods:**

We conducted a multinational cross-sectional study of interbirth interval (time between two singleton live births) and maternal anaemia (haemoglobin levels < 12 g/dl for non-pregnant women, < 11 g/dl for pregnant women) for 21 sub-Saharan African countries using the most recent nationally representative Demographic and Health Surveys, 2010–2017. A weighted multivariable fractional polynomial function was used to estimate the non-linear relationship between interbirth interval and maternal anaemia, considering interbirth interval as a continuous variable and adjusting for potential confounders. Analyses were stratified by reproductive classification (non-pregnant and pregnant women).

**Results:**

There were 81,693 women included in the study (89.2% non-pregnant, 10.8% pregnant). Of all women, 32.2% were in their postpartum period. Overall, 36.9% of women had anaemia (36.0% of non-pregnant and 44.3% of pregnant women). Of the participants, 15% had a short interbirth interval (<24 months), and 16% had a long interbirth interval (≥ 60 months). We found that both short and longer interbirth intervals were associated with an increased risk of maternal anaemia in a dose-response fashion. Relatively a lower risk of maternal anaemia was observed between 24 and 40 months of interbirth intervals.

**Conclusions:**

Our findings suggest that both short and longer interbirth intervals were associated with an increased risk of maternal anaemia in sub-Saharan Africa.

## Introduction

Anaemia is a condition in which haemoglobin concentration and red blood cell numbers are lower than normal and insufficient to meet an individual’s physiological needs [1]. Women of reproductive age are vulnerable to anaemia due to the physiological mechanisms of persistent menstrual blood loss and the demands of repeated pregnancy and childbearing [1]. Globally, anaemia was affected for approximately 39% of women of reproductive age, 33% of non-pregnant, and 40% of pregnant women in 2016 [2]. Women in Sub-Saharan Africa (SSA) suffer the highest-burden of anaemia, where 39% of women of reproductive age, 38% of non-pregnant women and 46% of pregnant women were affected by anaemia [2]. Maternal anaemia has been associated with increased maternal mortality and morbidity [3, 4] and adverse birth outcomes such as miscarriage, stillbirth, preterm birth, low birth weight and neonatal death [5].

The WHO defines a short interpregnancy interval (birth-to-pregnancy interval) as less than 24 months, which is approximately 33 months of interbirth interval (birth-to-birth interval)—the proxy measure of the interpregnancy interval [6]. Several studies have examined whether the interbirth interval is associated with an increased risk of maternal anemia [7–10]. However, the results of previous studies were inconsistent. Some studies found that the short interbirth interval is associated with increased maternal anaemia [7, 8, 11], while others found little evidence for a relationship between short interbirth interval and maternal anaemia [9, 10, 12, 13]. Although the biological mechanisms by which short interbirth intervals may lead to maternal anaemia have not been fully elucidated [14], it has been suggested that micronutrient depletion may partially explain associations between a short interbirth interval and maternal anaemia [15]. Pregnancy is associated with increased demand for nutrients, such as iron and folate, required to increase the red cell mass, expand the plasma volume, and allow for the growth of the foetus and uteroplacental organs [16]. Lactation presents a continuation of this demand [17]. A short interval between pregnancies provides insufficient time for women to replenish their nutritional reserves prior to subsequent pregnancies, which increases the risk of maternal anaemia and adverse pregnancy outcomes [18–21].

Longer interbirth intervals are thought to be protective against maternal anemia [9], due to avoidance of risk arising from short intervals. However, there is accumulating evidence that longer intervals, particularly those in the upper range (≥60 months), may increase risk [22]. Similar to short interbirth intervals, the specific mechanism by which long intervals cause maternal anemia is not well established. Because a biologically plausible pathway by which long interbirth intervals lead to maternal anaemia may be through its mediating effects on antepartum and postpartum haemorrhage and preeclampsia [22, 23].

Although most previous studies have focused on associations between short interbirth intervals and maternal anemia, they have shown conflicting results [7–13, 24–29]. Almost all previous studies examined its association by treating interbirth interval as categorical, most often as dichotomous, despite the inherent continuity of this exposure and the lack of evidence for specific discrete exposure thresholds [8, 24–26, 30]. The use of discretely classified interbirth intervals can lead to several issues, most notably information loss, which leads to reduced statistical power [31–33]. The aim of this study was to investigate the association between interbirth interval and maternal anaemia along the continuum of exposure. Identifying the association of interbirth interval and maternal anaemia in the SSA region, which has a higher fertility rate (high rate of the short interbirth interval) and burden of maternal anaemia, may provide further justification for promoting birth spacing using effective modern contraceptive methods, which in turn should decrease the burden of maternal anaemia and adverse pregnancy outcomes.

## Methods

### Study design

A cross-sectional study was employed to assess the association between maternal anaemia and interbirth intervals in SSA. We obtained data from the most recent Demographic and Health Surveys (DHS) conducted from 2010 to 2017.

### Study setting

Twenty-one SSA countries (eight Western Africa, six Southern Africa, four Eastern Africa and three Central Africa countries), which have haemoglobin concentration in their DHS dataset and are available in the Integrated Public Use Microdata Series (IPUMS)-DHS [34] were included in this study. The DHS is a representative household survey that employs a stratified two-stage cluster sampling design and is conducted every three to five years in participating countries. The standard DHS collects information on sociodemographic, maternal and child health and nutrition from women, under-five children and men. Details of the DHS collection and sampling procedure have been published previously [35].

### Participants

**Fig 1:**
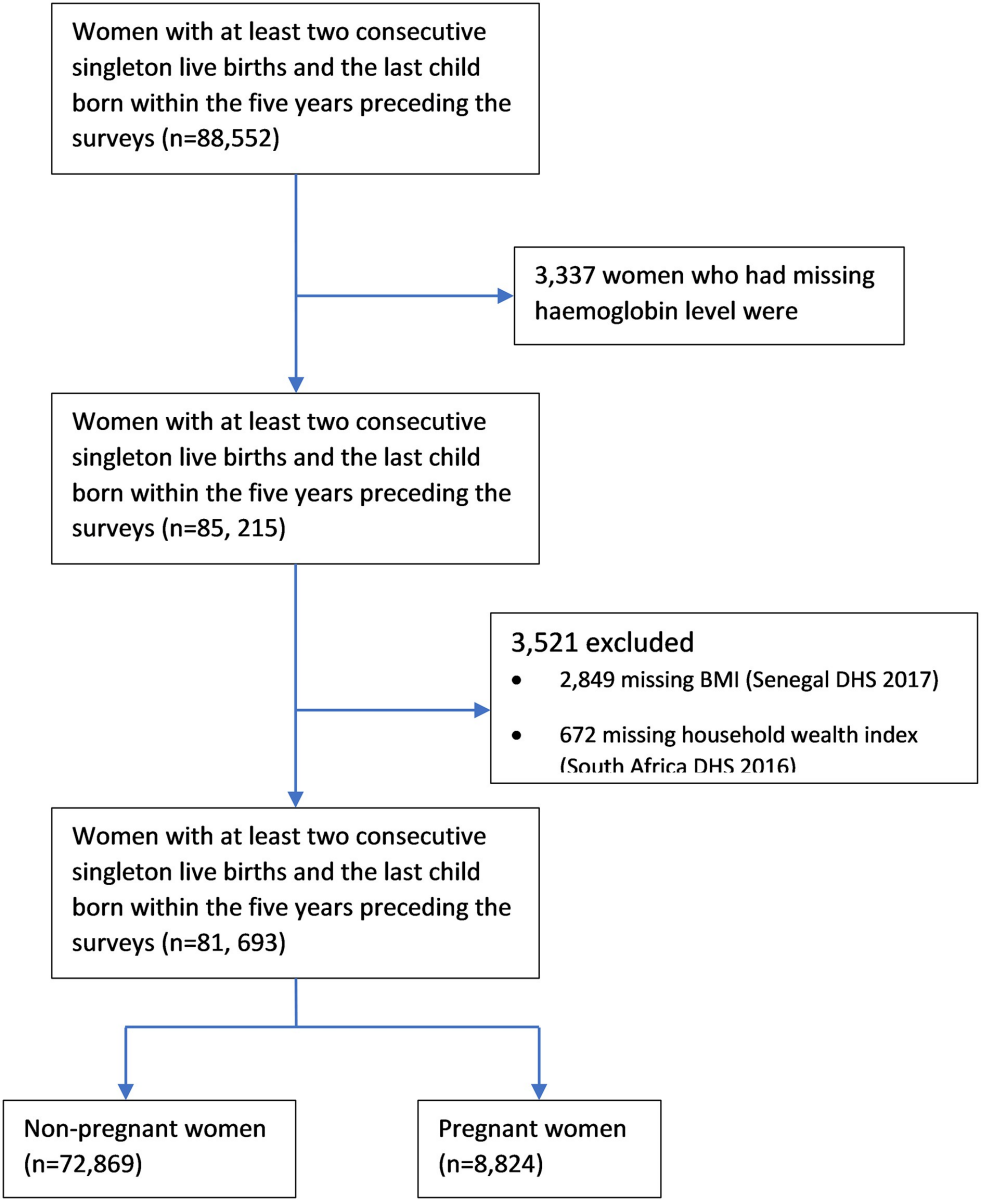
Flow chart of the analytic sample selection process.

In total, 88,552 women who had at least two consecutive singleton live births with the last child born within the five years preceding the survey were eligible for this study. Women with a missing haemoglobin level (n = 3,337) were excluded. Furthermore, due to a lack of data on maternal BMI (n = 2850) and household wealth index (n = 672), the Senegal DHS, 2017 and South Africa DHS, 2016 were not included in the final model. Finally, the analytical cohort includes a sample of 81,693 women. From this cohort, we defined the following sub-cohorts based on reproductive status: (i) women who were not pregnant at the time of the DHS interview (n = 72,869) and (ii) women who were pregnant at the time of the DHS interview (n = 8,824) [Fig 1].

### Variables

#### Outcome variable

The outcome variable was maternal anaemia, defined by haemoglobin concentrations of capillary blood. The haemoglobin concentration was adjusted for altitude based on WHO protocols [1]. Anaemia was defined in non-pregnant women when the haemoglobin concentration was less than 12 g/dl and in pregnant women when the haemoglobin concentration was less than 11 g/dl [1].

#### Exposure variable

The exposure variable was the interbirth interval, which is defined as the time elapsed between the birth date of the latest child and the date of birth of the preceding child expressed in months. Interpregnancy interval was computed for the pregnant women sub-cohort by subtracting the gestational months at the time of the survey from the time elapsed between the birth date of the last child and the date of the survey.

#### Covariates

Based on previous literature, sociodemographic and household characteristics and health service-related adjustment variables were identified. The adjustment variables were maternal age (15–19, 20–24, 25–29, 30+ years), maternal education (no education, primary, secondary or above), marital status (married vs not married/in union), employment status (working vs not working), maternal Body Mass Index (BMI) (underweight: <18 kg/m^2^; normal: 18.5–24.9 kg/m^2^; and overweight/obese: ≥25 kg/m^2^), parity (1, 2–4 and ≥5) previous history of pregnancy loss (yes vs no), place of residence (rural vs urban), household size (1–5 vs ≥ 6 people), wealth index, antenatal care (yes/no), iron supplementation (yes/no), place of delivery (home vs facility), caesarean-section delivery (yes/no), postnatal care (yes/no), breastfeeding status of last-child, current contraceptive use, the birth year of the last child, time from date of last birth to date of haemoglobin measurement and country. The breastfeeding status was categorised as breastfeeding, not breastfeeding and never breastfed, and the current contraceptive use was categorised as currently using, not using and pregnant. The household wealth index was computed by the principal component analysis using household assets, possessions, and amenities. The wealth index was already available in the DHS dataset as five quintiles ranked as poorest, poorer, middle, richer and richest. However, we re-categorised it into lowest (includes poorest and poorer), middle and highest (includes richer and richest) [36].

### Statistical analysis

We used a modified Poisson regression and modelled a multivariable fractional polynomial to estimate the non-linear relationship between interbirth interval and maternal anaemia, considering interbirth interval as a continuous variable. We fitted the first (FP1) and second-degree (FP2) fractional polynomial transformation with the power of the polynomial (-2, -1, -.5, 0, .5, 1, 2, 3) after adjusting for the potential confounders. Variables such as maternal age, household family size, parity, maternal BMI, the birth year of the last child and the interval from the birth of the last child to the measurement of haemoglobin, were adjusted as continuous; and variables such as household wealth index, women’s educational attainment, marital status, employment status, etc. were considered as a categorical variable. The best-fitted degree fractional polynomial was selected based on the lower value of Akakies Information Criteria (AIC). The analyses were carried out across the full length of the interbirth interval. We assessed an interaction effect between interbirth interval and country using a likelihood-ratio test. All the analyses accounted for sampling weight and the complex sampling design and were conducted using Stata 16.1 [37].

### Sensitivity analysis

Maternal anaemia levels may vary across the different reproductive periods (pregnancy and non-pregnant) due to biological mechanisms and /or obstetric complications. To examine if our results were sensitive to the reproductive periods, we conducted separate analyses for non-pregnant and pregnant women sub-cohorts separately. The frequency table was used to present the characteristics of women in the analytical cohort and all eligible women.

### Ethical considerations

Since the DHS is ethically approved in each country, this study’s analysis was carried out without the need for ethical review after getting access to the DHS’s data.

## Results

### Cohort description

Of the 88,552 eligible participants, 81,693 (92.3%) were included in the analytical cohort (Fig 1). The women’s characteristics were similar across the analytical cohort and the eligible women (Table 1 and S1 Table). The majority of women were married (90%) and rural residents (78%). The mean age of women at the last child’s birth was 29 years (SD ± 6.4). Non-educated and women who had the lowest household wealth index were 43% and 44%, respectively. Of the participating women, 15% had a short interbirth interval (<24 months) and 16% had a long interbirth interval (≥ 60 months). Twenty-two per cent of pregnant women and fourteen per cent of non-pregnant women had a short interbirth interval (Table 1).

**Table 1:** Sociodemographic, household and health services characteristics of the participant women in the analysis population (n = 81,693) in sub-Saharan Africa 2010–2017.

Variable	Category	Frequency	Percentage
Current pregnancy status	Non-pregnant	72,869	89.2
	Pregnant	8,824	10.8
Maternal age at birth of the last child in years	15–19	2,688	3.3
	20–24	17,925	21.9
	25–29	24,079	29.5
	≥30	37,002	45.3
Maternal educational status	No education	34,909	42.7
	Primary	33,049	40.5
	Secondary and above	13,733	16.8
Marital status	Married	73,385	89.8
	Not married/in union	8,307	10.2
Maternal employment status	Not employed	31,934	39.1
	Employed	49,703	60.8
	Missing	56	0.07
Parity	1	19,858	24.3
	2–4	39,799	48.7
	≥5	22,036	27.0
Interbirth interval in months	<24	12,453	15.2
	24–35	27,445	33.6
	36–47	18,336	22.4
	48–59	9,940	12.2
	≥60	13,519	16.5
Maternal BMI (k.g/m^2^)	Underweight	8,024	9.8
	Normal	57,158	70.0
	Overweight	16,512	20.2
Household size	1–5 people	34,574	42.3
	≥6 people	47,120	57.7
Place of residence	Urban	18,089	22.1
	Rural	63,604	77.9
Wealth index	Lowest	36,191	44.3
	Middle	16,794	20.6
	Highest	28,709	35.1
Past history of pregnancy loss	No	69,290	84.8
	Yes	12,390	15.2
Antenatal care during pregnancy of the last child	No	10,912	13.4
	Yes	70,710	86.6
	Missing	71	0.09
Iron supplementation during pregnancy of last child	No	25,008	30.6
	Yes	56,356	69.0
	Missing	329	0.4
Place of delivery for last child	Home	32,115	39.3
	Health facility	48,140	58.9
	Missing	1,438	1.8
Caesarean-section delivery of last-child	No	78,182	95.7
	Yes	3,389	4.2
	Missing	123	0.2
Postnatal care for the last child	No	56,907	69.7
	Yes	24,679	30.2
	Missing	107	0.1
Current contraceptive use	No	45,454	55.6
	Yes	27,415	33.6
	Pregnant	8,824	10.8
Breastfeeding status of the last child	Not breastfeeding	30,159	36.9
	Still breastfeeding	49,487	60.6
	Never breastfed	1,904	2.3
	Missing	143	0.2

### Anaemia prevalence among sub-Saharan African women

In this study, the prevalence of maternal anaemia was 36.9% (36.0% of non-pregnant and 44.3% of pregnant women). Anaemia was observed among 42.1% of women who had no education. Similarly, 42.1% of women 15–19 years of age had anaemia. The prevalence of anaemia was higher among women who had six or more household sizes (38.6%) and lowest household wealth index (39.2%) than women with 1–5 household sizes (34.7%) and highest household wealth index (34.2%). Forty-two per cent of women who were not using any contraceptive had anaemia compared to women who were using (26.4%). However, no difference was observed in the prevalence of anaemia among women living in rural and urban residences and among employed and not employed women (Table 2 and S2 Table). As the date of the last birth to the date of haemoglobin measurement increases, the maternal anaemia level decreases (42.5% at six months, vs 34% at 48–60 months) (S1 Fig). Generally, Western Africa had the highest maternal anaemia (49.7%) of the SSA regions. The prevalence of maternal anaemia was higher in Cote d’Ivoire, Guinea, Mali, Senegal and Burkina Faso. However, except in Mozambique, the Southern African Countries had the lowest maternal anaemia prevalence (S3 Table).

**Table 2:** The characteristics of the participants in the analysis population by maternal anaemia in sub-Saharan Africa 2010–2017.

Variable	All women (n = 81,693)		Non-pregnant (n = 72,869)		Pregnant (n = 8,824)	
	Non-anaemic (n = 51,526)	Anaemic (n = 30,167)	Non-anaemic (n = 46,607)	Anaemic (n = 26,262)	Non-anaemic (n = 4,919)	Anaemic (n = 3,905)
**Maternal age at birth of the preceding child**						
15–19 years	1,556 (57.9)	1,132 (42.1)	1,315 (59.0)	914 (41.0)	241 (52.4)	218 (47.6)
20–24 years	11,311 (63.1)	6,613 (36.9)	9,875 (64.0)	5,556 (36.0)	1,437 (57.6)	1,057 (42.4)
25–29 years	15,523 (64.5)	8,556 (35.5)	13,884 (65.6)	7,276 (34.4)	1,639 (56.2)	1,280 (43.8)
≥30 years	23,136 (62.5)	13,866 (37.5)	21,533 (63.2)	12,516 (36.8)	1,603 (54.3)	1,350 (45.7)
**Maternal education**						
No education	20,220 (57.9)	14,689 (42.1)	18,048 (58.9)	12,601 (41.1)	2,172 (51.0)	2,087 (49.0)
Primary	21,864 (66.2)	11,185 (33.8)	19,752 (66.9)	9,768 (33.1)	2,112 (59.9)	1,417 (40.1)
Secondary and above	9,440 (68.7)	4,293 (31.3)	8,806 (69.3)	3,892 (30.7)	634 (61.3)	401 (38.7)
**Maternal BMI in k.g/m** ^**2**^						
Underweight	4,815 (60.0)	3,209 (40.0)	4,574 (60.0)	3,054 (40.0)	241 (60.8)	156 (39.2)
Normal	35,528 (62.2)	21,630 (37.8)	32,044 (63.1)	18,719 (36.9)	3,484 (54.5)	2,910 (45.5)
Overweight	11,184 (67.7)	5,328 (32.3)	9,989 (69.0)	4,489 (31.0)	1,194 (58.7)	839 (41.3)
**Maternal employment**						
Not working	20,202 (63.3)	11,732 (36.7)	18,108 (64.3)	10,046 (35.7)	2,094 (55.4)	1,686 (44.6)
Working	31,294 (63.0)	18,409 (37.0)	28,471 (63.7)	16,196 (36.3)	2,823 (56.1)	2,213 (43.9)
Missing	30 (54.2)	26 (45.8)	29 (58.9)	20 (41.2)	2 (24.3)	6 (75.7)
**Marital status**						
Married	46,415 (63.2)	26,970 (36.8)	41,668 (64.2)	23,231 (35.8)	4,747 (55.9)	3,739 (44.1)
Not married/in union	5,111 (61.5)	3,196 (38.5)	4,939 (62.0)	3,031 (38.0)	172 (51.0)	165 (49.0)
**Parity**						
1	12,819 (64.5)	7,040 (35.5)	11,443 (65.3)	6,091 (34.7)	1,376 (59.2)	949 (40.8)
2–4	25,321 (63.6)	14,478 (36.4)	22,804 (64.6)	12,479 (35.4)	2,517 (55.7)	1,999 (44.3)
≥5	13,387 (60.7)	8,649 (39.3)	12,360 (61.6)	7,692 (38.4)	1,027 (51.7)	1957 (48.3)
Interbirth interval in months						
<24 months	7,795 (62.6)	4,659 (37.4)	6,700 (63.7)	3,816 (36.3)	1,095 (56.5)	843 (43.5)
24–35 months	16,886 (61.5)	10,559 (38.5)	14,950 (62.7)	8,912 (37.3)	1,936 (54.0)	1,647 (46.0)
36–47 months	11,487 (62.5)	6,850 (37.4)	10,454 (63.5)	6,021 (36.5)	1,032 (55.5)	829 (44.5)
48–59 months	6,419 (64.6)	3,521 (35.4)	5,975 (65.2)	3,192 (34.8)	444 (57.4)	329 (42.6)
≥60 months	8,940 (66.1)	4,579 (33.9)	8,529 (66.4)	4,321 (933.6)	411 (61.5)	258 (38.5)
**Household size**						
1–5 people	22,578 (65.3)	11,996 (34.7)	20,151 (66.1)	10,318 (33.9)	2,427 (59.1)	1,678 (40.9)
≥6 people	28,949 (61.4)	18,171 (38.6)	26,456 (62.4)	15,944 (37.6)	2,492(52.8)	2,227 (47.2)
**Residence**						
Urban	11,420 (63.1)	6,670 (36.9)	10,578 (63.8)	6,010 (36.2)	842 (56.1)	660 (43.9)
Rural	40,107 (63.1)	23,498 (36.9)	36,030 (64.0)	20,252 (36.0)	4,077 (55.7)	3,245 (44.3)
**Wealth index**						
Lowest	22,001 (60.8)	14,190 (39.2)	19,633 (61.6)	12,241 (38.4)	2,368 (54.9)	1,949 (45.1)
Middle	10,625 (63.3)	6,169 (36.70	9,597 (64.2)	5,353 (35.8)	1,028 (55.8)	816 (44.2)
Highest	18,900 (65.8)	9,809 (34.2)	17,377 (66.7)	8,668 (33.3)	1,523 (57.2)	1,141 (42.8)
Past history of pregnancy loss						
No	43,895 (63.3)	25,395 (36.7)	39,817 (64.3)	22,079 (35.7)	4,078 (55.2)	3,316 (44.8)
Yes	7,623 (61.5)	4,767 (38.5)	6,787 (61.9)	4,179 (38.1)	836 (58.7)	588 (41.3)
**Antenatal care**						
No	7,055 (64.7)	3,857 (35.3)	6,215 (65.6)	3,264 (34.4)	840 (58.6)	593 (41.4)
Yes	44,432 (62.8)	26,278 (37.2)	40,358 (63.7)	22,976 (36.3)	4,073 (55.2)	3,302 (44.8)
Missing	39 (55.5)	32 (44.5)	35 (60.9)	22 (39.1)	5 (34.5)	9 (65.5)
**Iron supplementation**						
No	16,206 (64.8)	8,802 (35.2)	14,474 (65.7)	7,549 (34.3)	1,732 (58.0)	1,253 (42.0)
Yes	35,121 (62.3)	21,236 (37.7)	31,953 (63.2)	18,606 (36.8)	3,168 (54.6)	2,629 (45.4)
Missing	199 (60.6)	130 (39.4)	181 (62.9)	106 (37.1)	19 (44.6)	23 (55.4)
**Place of delivery**						
Home	19,793 (61.6)	12,322 (38.4)	17,551 (62.8)	10,386 (37.2)	2,242 (53.7)	1,936 (46.3)
Health facility	30,861 (64.1)	17,278 (35.9)	28,270 (64.8)	15,363 (35.2)	2,592 (57.5)	1,915 (42.5)
Missing	871 (60.6)	567 (39.4)	786 (60.5)	513 (39.5)	85 (61.2)	54 (38.8)
**Caesarean-section delivery**						
No	49,183 (62.9)	28,999 (37.1)	44,412 (63.8)	25,182 (36.2)	4,771 (55.6)	3,817 (44.4)
Yes	2,269 (67.0)	1,120 (33.0)	2,127 (67.2)	1,037 (32.8)	142 (63.2)	83 (36.8)
Missing	74 (60.3)	49 (39.7)	68 (61.5)	43 (38.5)	6 (50.)	6 (50.0)
**Postnatal care**						
No	36,395 (64.0)	20,513 (36.0)	32,765 (65.0)	17,680 (35.0)	3,630 (56.2)	2,832 (43.8)
Yes	15,060 (61.0)	9,619 (39.0)	13,775 (61.7)	8,552 (38.3)	1,285 (54.6)	1,067 (45.4)
Missing	71 (66.7)	36 (33.3)	67 (69.1)	30 (30.9)	4 (44.1)	6 (55.9)
Still breastfeeding	31,397 (63.4)	18,090 (36.6)	30,348 (63.6)	17,371 (36.4)	1,049 (59.4)	718 (40.6)
Not breastfeeding	18,899 (62.7)	11,261 (37.3)	15,212 (64.9)	8,215 (35.1)	3,687 (54.8)	3,046 (45.2)
Never breastfeeding	1,148 (60.3)	756 (39.7)	980 (61.0)	626 (39.0)	168 (56.4)	130 (43.6)
Missing	82 (57.5)	61 (42.5)	67 (57.4)	50 (42.6)	15 (58.3)	11 (41.7)
**Current contraceptive use**						
Not using	26,431 (58.1)	19,023 (41.9)	26,431 (58.1)	19,023 (41.9)	-	-
Using	20,177 (73.6)	7,239 (26.4)	20,177 (73.6)	7,239 (26.4)	-	-
Pregnant	4,919 (55.7)	3,905 (44.3)	-	-		
**Time from last birth to date of interview (Mean±SD)**	23.2±15.6	21.9±15.8	22.2±15.6	20.5±15.8	32.2±11.7	31.7±11.6

### Association between interbirth interval and maternal anaemia

**Fig 2:**
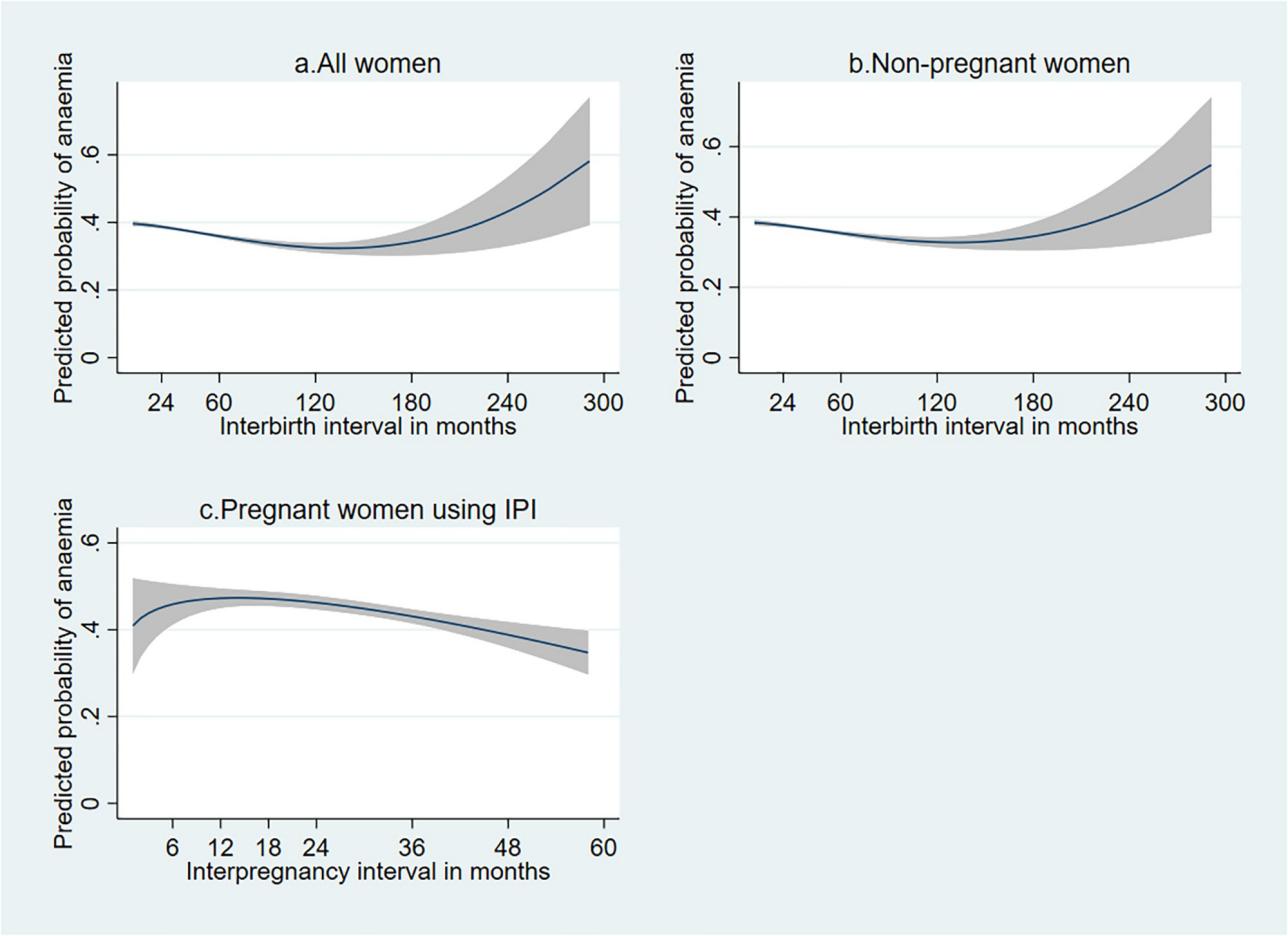
The association between interbirth interval and predicted maternal anaemia based on the first-degree fractional polynomial.

The multivariable first-degree fractional polynomial showed that short and very long (>120 months) interbirth intervals increased the risk of maternal anaemia. The risk of maternal anaemia slightly declined as the interbirth interval increased, which sharply increased at approximately 120 months. The sensitivity analysis among pregnant and non-pregnant women showed consistent results. Additionally, despite using the interpregnancy interval limited to less than 60 months due to the study participants who gave their last birth during the five years preceding the survey, the sensitivity analysis among pregnant women also showed a similar pattern (Fig 2).

These graphs showed the relationship between interbirth interval and predicted maternal anaemia among different groups of cohorts using the first-degree fractional polynomial. The y-axis represents the predicted risk of maternal anaemia, whereas the x-axis represents the interbirth interval in months (graphs a and b) and interpregnancy interval in months in graph c. The black lines denote the predicted risk of maternal anaemia, and the shaded regions denote a 95% confidence interval for the fractional polynomial. In graph a and b, adjustment was made for maternal age at birth of the last-child (continuous), maternal education, employment status, marital status, residence, household wealth index, time from last birth to date of haemoglobin measurement (continuous), antenatal care, iron supplementation, place of delivery, caesarean-section delivery, postnatal care, breastfeeding status and current contraceptive use, country, parity, maternal BMI (continuous) and household size (continuous). In graph c, adjustment was made for all the above-listed variables except for breastfeeding status and current contraceptive use, as pregnant women are not eligible.

## Discussion

This study examined the association between interbirth interval and maternal anaemia using DHS data from 21 SSA countries conducted between 2010 and 2017. We found an increased risk of maternal anaemia with both short and very long interbirth intervals. A similar pattern was also observed in the sensitivity analysis among the sub-cohorts of women. Our finding is consistent with the previous studies that indicated that a short interbirth interval increases the risk of anemia [7, 8, 11, 24–28]. In contrast, some studies showed that a short interbirth interval has no association with maternal anaemia [9, 10, 12, 13]. The majority of the previous studies modelled interbirth interval as a categorical variable in regression cannot capture the non-linear relationship between interbirth interval and many perinatal outcomes and may produce a biased estimate of the association [38, 39].

The WHO recommendation on birth spacing suggests that women wait a minimum of 24 months before attempting subsequent pregnancy, but the maximum limit at which women/couples can stay without getting a subsequent pregnancy is unclear [6]. The focus on a short interbirth interval might be due to the high level of fertility and the lack of evidence on the impacts of the long interbirth intervals. However, the current study demonstrated that long interbirth intervals increased maternal anaemia more than short interbirth intervals. Therefore, in addition to improving short interbirth intervals, it is also essential to educate and counsel women in SSA to reduce longer interbirth intervals.

The mechanism for the observed associations is not clear, but previous studies hypothesise that short interbirth intervals may be related to maternal depletion [40, 41]. Normally, energy need is increased during pregnancy [42]. If women received nutrient supply inadequate to meet the needs for pregnancy, women’s nutritional reserves would be depleted [21]. Women who conceive within a short interbirth interval might not get adequate time to replenish their depleted reserves during the preceding pregnancy, resulting in maternal anaemia [41, 43]. The physiological regression hypothesis might explain the association between long interbirth intervals and adverse perinatal outcomes [41, 44]. When women conceive their subsequent pregnancy after long intervals, their body mechanism returns to the primigravida state, strongly associated with most adverse pregnancy outcomes likely to result in the risk of maternal anaemia [41]. Our study is based on a cross-sectional, in which the confounding variables were collected at a time, and some expected confounders variables were not measured; we suggested a further longitudinal study on the role of interbirth interval on the risk of maternal.

This study strength is that the collation of surveys of low-income SSA countries resulted in a large sample size. Unlike previous studies, this study used a fractional polynomial function to account for the non-linear relationship between interbirth interval and maternal anemia. As shown in our supplementary analysis, categorising the interbirth interval could lead to a biased estimate as information might be missed due to the categorisation of the interbirth interval. However, the following limitations should be considered when interpreting our findings. Due to the cross-sectional nature of the data collection, there was variability in the length of time between haemoglobin measurement and the date of delivery of the last birth. Women included in the study gave birth to their last child sometime in the five years preceding the surveys; however, the haemoglobin was measured at the time of the survey; therefore, women who had given birth in the earliest period would have the opportunity to replenish their depleted nutrient stores in the intervening period. However, to overcome this limitation, we have adjusted for the time between the birth date of the last-child and haemoglobin measurement. Despite the fact that previous successive interbirth intervals might have a cumulative effect on maternal anaemia in mothers with more than two births, in this study, we considered only the interbirth interval between the most recent and preceding birth. A further limitation was that the interbirth interval was self-reported, and there was a potential for recall bias. As the DHS surveys do not provide information on the gestational age of the last birth, it was not possible to directly estimate the interpregnancy interval, i.e., the time between preceding birth and conception of the last birth. Instead, the interbirth interval was used as its proxy. A similar approach has been used in several studies that lacked information on gestational age [29, 45, 46]. A number of variables, including chronic illness, pregnancy complications, parasitic infections and maternal dietary intake, cultural norms, values and beliefs, which might affect either the interbirth intervals or maternal anaemia, are not available in the DHS dataset. Therefore, we were unable to adjust for these likely confounding variables in our analyses. Additionally, the health services factors that have been adjusted for as confounders were proxy variables. These variables were measured after the conception of the last birth, which might not affect the interbirth interval. Finally, as not all SSA countries have conducted a DHS and the DHS data are not available in the IPUMS-DHS for a few countries which have conducted a DHS, we cannot generalise our findings to all SSA countries.

## Conclusions

Our finding suggests that both short and longer interbirth intervals were associated with the risk of maternal anaemia in SSA. Therefore, to prevent maternal anaemia in SSA, the governments should emphasise both short and long birth spacing in their policies and strategies, and health care providers should also advise women not to conceive early and delay subsequent pregnancies.

## Supporting information

S1 Fig The prevalence of maternal anaemia by time. (TIFF)

S1 Table Sociodemographic, household and health services characteristics of all eligible participants. (DOCX)

S2 Table The characteristics of eligible participant women by maternal anaemia. (DOCX)

S3 Table The number of samples included and maternal anaemia level. (DOCX)

S1 Appendix STROBE 2007 (v4) statement—Cross-sectional checklist. (DOCX)
