# Supplementary figures and images for: Interbirth interval and maternal anaemia in 21 sub-Saharan African countries: A fractional-polynomial analysis
Source: PLoS One. 2022 Sep 23;17(9):e0275155. doi: 10.1371/journal.pone.0275155 (PMC9506648; doi:10.1371/journal.pone.0275155)

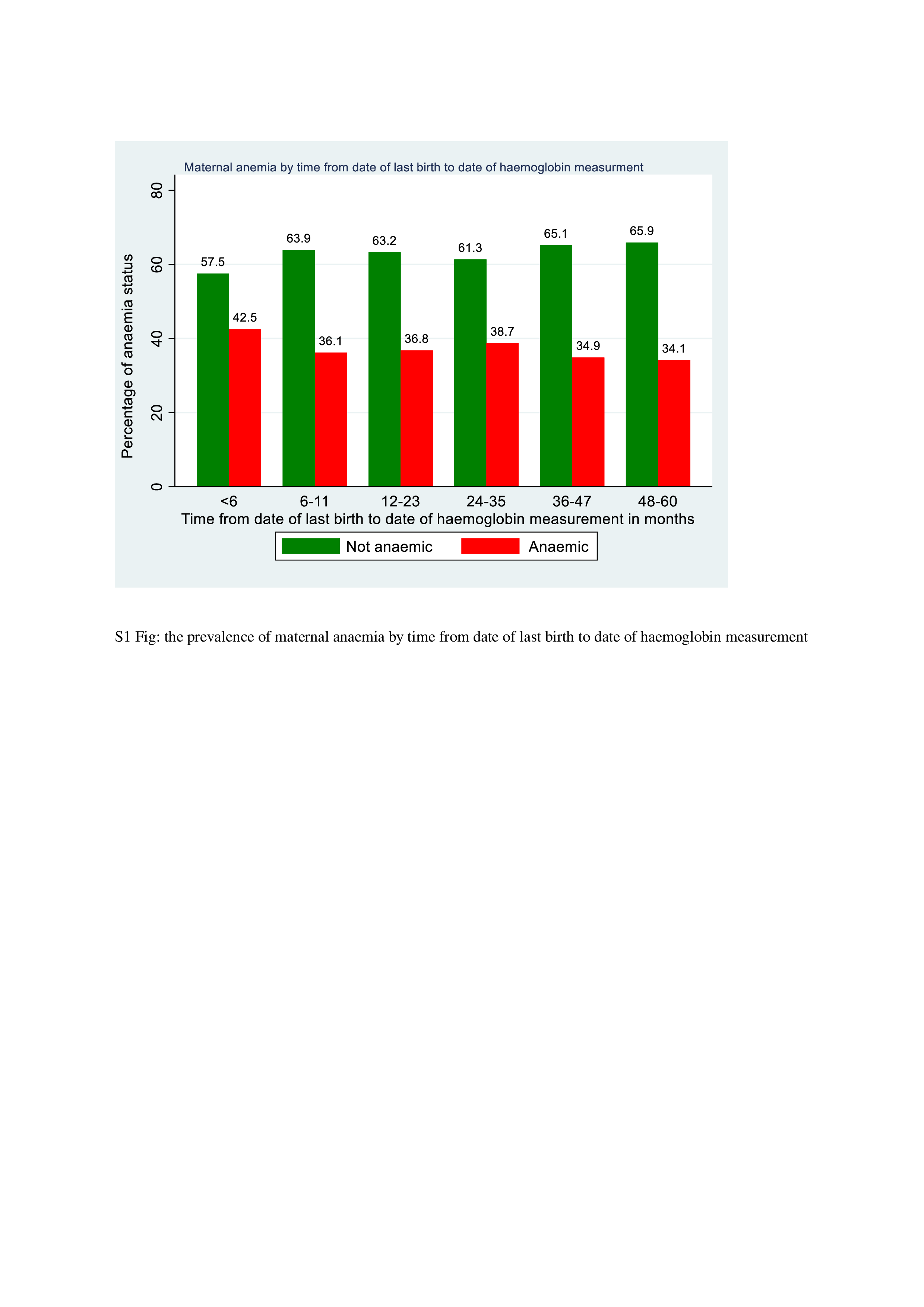

Supplement: S1 Fig — (TIFF) [file pone.0275155.s001.tiff]
