# Supplementary material for: Interbirth interval and maternal anaemia in 21 sub-Saharan African countries: A fractional-polynomial analysis
Source: PLoS One. 2022 Sep 23;17(9):e0275155. doi: 10.1371/journal.pone.0275155 (PMC9506648; doi:10.1371/journal.pone.0275155)
Supplement: S1 Table — (DOCX) [file pone.0275155.s002.docx]

S1 Table. Sociodemographic, household and health services characteristics of all eligible participant women (n= 88,552) in Sub-Saharan Africa 2010-2017.

| **Variable** | **Category** | **Frequency** | **Percentage** |
| --- | --- | --- | --- |
| Current pregnancy status | Non-pregnant | 79,117 | 89.3 |
|  | Pregnant | 9,435 | 10.7 |
| Maternal age at birth of the last child in years | 15-19 | 2,901 | 3.3 |
|  | 20-24 | 19,247 | 21.7 |
|  | 25-29 | 26,037 | 29.4 |
|  | ≥30 | 40,367 | 45.6 |
| Maternal educational status | No education | 38,049 | 43.0 |
|  | Primary | 34,823 | 39.3 |
|  | Secondary and above | 15,678 | 17.7 |
| Marital status | Married | 79,358 | 89.6 |
|  | Not married/in union | 9,193 | 10.4 |
| Maternal employment status | Not employed | 35,255 | 39.8 |
|  | Employed | 53,238 | 60.1 |
|  | Missing | 59 | 0.07 |
| Parity | 1 | 21,854 | 24.7 |
|  | 2-4 | 43,162 | 48.7 |
|  | ≥5 | 23,536 | 26.6 |
| Interbirth interval in months | <24 | 13,423 | 15.2 |
|  | 24-35 | 29,628 | 33.5 |
|  | 36-47 | 19,726 | 22.3 |
|  | 48-59 | 10,825 | 12.2 |
|  | ≥60 | 14,951 | 16.9 |
| Maternal BMI (k.g/m^2^) | Underweight | 8,159 | 9.2 |
|  | Normal | 58,308 | 65.8 |
|  | Overweight | 19,085 | 21.6 |
|  | Missing | 3,001 | 3.4 |
| Household size | 1-5 people | 36,672 | 41.4 |
|  | ≥6 people | 51,881 | 58.6 |
| Place of residence | Urban | 20,890 | 23.6 |
|  | Rural | 67,663 | 76.4 |
| Wealth index | Lowest | 38,634 | 43.6 |
|  | Middle | 17,929 | 20.2 |
|  | Highest | 31,029 | 33.0 |
|  | Missing | 960 | 1.1 |
| Past history of pregnancy loss^[[1]](#footnote-1)^ | No | 72,084 | 84.6 |
|  | Yes | 13,119 | 15.4 |
| Antenatal care during pregnancy of the last child | No | 11,622 | 13.1 |
|  | Yes | 76,855 | 86.8 |
|  | Missing | 76 | 0.08 |
| Iron supplementation during pregnancy of last-child | No | 26,227 | 29.6 |
|  | Yes | 61,970 | 70.0 |
|  | Missing | 355 | 0.4 |
| Place of delivery for last-child | Home | 33,931 | 38.3 |
|  | Health facility | 53,061 | 59.9 |
|  | Missing | 1,561 | 1.8 |
| Caesarean-section delivery of last-child | No | 84,530 | 95.5 |
|  | Yes | 3,896 | 4.4 |
|  | Missing | ­­127 | 0.1 |
| Postnatal care for the last child | No | 60,187 | 68.0 |
|  | Yes | 28,254 | 31.9 |
|  | Missing | 112 | 0.1 |
| Current contraceptive use | No | 49,115 | 55.5 |
|  | Yes | 30,002 | 33.9 |
|  | Pregnant | 9,435 | 10.7 |
| Breastfeeding status of last child | Not breastfeeding | 32,773 | 37.0 |
|  | Still breastfeeding | 53,379 | 60.3 |
|  | Never breastfed | 2,234 | 2.5 |
|  | Missing | 166 | 0.2 |

1. Pregnancy loss includes miscarriage or spontaneous abortion, and stillbirth [↑](#footnote-ref-1)
