# Supplementary material for: Interbirth interval and maternal anaemia in 21 sub-Saharan African countries: A fractional-polynomial analysis
Source: PLoS One. 2022 Sep 23;17(9):e0275155. doi: 10.1371/journal.pone.0275155 (PMC9506648; doi:10.1371/journal.pone.0275155)
Supplement: S2 Table — (DOCX) [file pone.0275155.s003.docx]

S2 Table. The characteristics of eligible participant women by maternal anaemia in Sub-Saharan Africa 2010-2017

| Variable | All women^[[1]](#footnote-1)^ (n=85,215) | | Non-pregnant ^[[2]](#footnote-2)^(n=76,087) | | Pregnant ^[[3]](#footnote-3)^(n=9,128) | |
| --- | --- | --- | --- | --- | --- | --- |
|  | Non- anaemic (n=53,341) | Anaemic (n=31,874) | Non- anaemic (n=48,298) | Anaemic (n=27,789) | Non- anaemic (n=5,043) | Anaemic (n=4,085) |
| **Maternal age at birth of the preceding child** |  |  |  |  |  |  |
| 15-19 years | 1,603 (57.6) | 1,182 (42.4) | 1,361 (58.9) | 949 (41.1) | 242 (51.0) | 233 (49.0) |
| 20-24 years | 11,612 (62.7) | 6,914 (37.3) | 10,155 (63.6) | 5,814 (36.4) | 1,457 (57.0) | 1,100 (43.0) |
| 25-29 years | 16,041 (64.0) | 9,027 (36.0) | 14,351 (65.1) | 7,684 (34.9) | 1,690 (55.7) | 1,343 (44.3) |
| ≥30 years | 24,085 (62.0) | 14,751 (38.0) | 22,431 (62.7) | 13,342 (37.3) | 1,654 (54.0) | 1,409 (46.0) |
| **Maternal education** |  |  |  |  |  |  |
| No education | 21,030 (57.2) | 15,724 (42.8) | 18,786 (58.2) | 13,506 (41.8) | 2,243 (50.3) | 2,219 (49.7) |
| Primary | 22,281 (65.9) | 11,547 (34.1) | 20,134 (66.6) | 10,093 (33.4) | 2,148 (59.6) | 1,454 (40.4) |
| Secondary and above | 10,028 (68.5) | 4,602 (31.5) | 9,376 (69.1) | 4,190 (30.9) | 652 (61.3) | 412 (38.7) |
| **Maternal BMI in k.g/m^2^** |  |  |  |  |  |  |
| Underweight | 4,824 (60.0) | 3,219 (40.0) | 4,583 (59.9) | 3,063 (40.1) | 241 (60.8) | 156 (39.2) |
| Normal | 35,646 (62.2) | 21,689 (37.8) | 32,154 (63.1) | 18,775 (36.9) | 3,490 (54.5) | 2,914 (45.5) |
| Overweight | 11,525 (67.8) | 5,465 (32.2) | 10,325 (69.1) | 4,625 (30.9) | 1,200 (58.8) | 841 (41.2) |
| Missing | 1,348 (47.3) | 1,501 (52.7) | 1,236 (48.2) | 1,326 (51.8) | 112 (39.0) | 175 (61.0) |
| **Maternal employment** |  |  |  |  |  |  |
| Not working | 21,114 (62.8) | 12,504 (37.2) | 18,953 (63.8) | 10,731 (36.2) | 2,161 (54.9) | 1,773 (45.1) |
| Working | 32,197 (62.5) | 19,344 (37.5) | 29,316 (63.2) | 17,038 (36.8) | 2,881 (55.5) | 2,306 (44.5) |
| Missing | 30 (54.2) | 26 (45.8) | 29 (58.8) | 20 (41.2) | 2 (24.3) | 6 (75.7) |
| **Marital status** |  |  |  |  |  |  |
| Married | 47,949 (62.7) | 28,509 (37.3) | 43,083 (63.7) | 24,595 (36.3) | 4,867 (55.4) | 3,913 (44.6) |
| Not married/in union | 5,392 (61.6) | 3,364 (38.4) | 5,215 (62.0) | 3,194 (38.0) | 176 (50.9) | 170 (49.1) |
| **Parity** |  |  |  |  |  |  |
| 1 | 13,395 (64.2) | 7,459 (35.8) | 11,989 (65.0) | 6,466 (35.0) | 1,405 (58.6) | 993 (41.4) |
| 2-4 | 26,194 (63.1) | 15,312 (36.9) | 23,607 (64.1) | 13,218 (35.9) | 2,587 (55.3) | 2,094 (44.7) |
| ≥5 | 13,752 (60.2) | 9,103 (39.8) | 12,701 (61.0) | 8,105 (39.0) | 1,051 (51.3) | 997 (48.7) |
| **Interbirth interval in months** |  |  |  |  |  |  |
| <24 months | 8,054 (62.1) | 4,920 (37.9) | 6,937 (63.2) | 4,035 (36.8) | 1,117 (55.8) | 884 (44.2) |
| 24-35 months | 17,453 (61.0) | 11,117 (39.0) | 15,458 (62.0) | 9,455 (38.0) | 1,995 (53.7) | 1,722 (46.3) |
| 36-47 months | 11,835 (62.2) | 7,193 (37.8) | 10,781 (63.0) | 6,320 (37.0) | 1,054 (54.7) | 873 (45.3) |
| 48-59 months | 6,670 (64.2) | 3,719 (35.8) | 6,212 (64.8) | 3,381 (35.2) | 458 (57.6) | 337 (42.4) |
| ≥60 months | 9,328 (65.7) | 4,866 (34.3) | 8,910 (66.0) | 4,597 (34.0) | 418 (60.9) | 269 (39.1) |
| **Household size** |  |  |  |  |  |  |
| 1-5 people | 22,994 (65.3) | 12,236 (34.7) | 20,552 (66.1) | 10,541 (33.9) | 2,442 (59.0) | 1,695 (41.0) |
| ≥6 people | 30,347 (60.7) | 19,638 (39.3) | 27,746 (61.7) | 17,248 (38.3) | 2,601 (52.1) | 2,390 (47.9) |
| **Residence** |  |  |  |  |  |  |
| Urban | 12,292 (62.8) | 7,287 (37.2) | 11,401 (63.4) | 6,589 (36.6) | 891 (56.1) | 698 (43.9) |
| Rural | 41,049 (62.5) | 24,587 (37.5) | 36,896 (63.5) | 21,200 (36.5) | 4,152 (55.1) | 3,387 (44.9) |
| **Wealth index** |  |  |  |  |  |  |
| Lowest | 22,538 (60.1) | 14,966 (39.9) | 20,112 (60.9) | 12,900 (39.1) | 2,426 (54.0) | 2,067 (46.0) |
| Middle | 10,930 (62.8) | 6,468 (37.2) | 9,880 (63.7) | 5,623 (36.3) | 1,050 (55.4) | 845 (44.6) |
| Highest | 19,406 (65.5) | 10,234 (34.5) | 17,852 (66.3) | 9,066 (33.7) | 1,554 (57.1) | 1,168 (42.9) |
| Missing | 467 (69.4) | 205 (30.6) | 455 (69.4) | 201 (30.6) | 12 (72.6) | 5 (27.4) |
| **Past history of pregnancy loss** |  |  |  |  |  |  |
| No | 45,348 (62.9) | 26,736 (37.1) | 41,180 (63.9) | 23,282 (36.1) | 4,167 (54.7) | 3,454 (45.3) |
| Yes | 7,985 (60.9) | 5,133 (39.1) | 7,114 (61.2) | 4,504 (38.8) | 871 (58.0) | 629 (42.0) |
| **Antenatal care** |  |  |  |  |  |  |
| No | 7,097 (64.5) | 3,911 (35.5) | 6,255 (65.4) | 3,312 (34.6) | 842 (58.4) | 599 (41.6) |
| Yes | 46,204 (62.3) | 27,931 (37.7) | 42,008 (63.2) | 24,455 (36.8) | 4,196 (54.7) | 3,476 (45.3) |
| Missing | 39 (55.5) | 32 (44.5) | 35 (60.9) | 22 (39.1) | 5 (34.5) | 9 (65.5) |
| **Iron supplementation** |  |  |  |  |  |  |
| No | 16,277 (64.7) | 8,889 (35.3) | 14,540 (65.6) | 7,626 (34.4) | 1,737 (57.9) | 1,263 (42.1) |
| Yes | 36,860 (61.7) | 22,855 (38.3) | 33,572 (62.6) | 20,056 (37.4) | 3,288 (54.0) | 2,799 (46.0) |
| Missing | 204 (61.7) | 130 (38.9) | 186 (63.4) | 107 (36.6) | 19 (44.6) | 23 (55.4) |
| **Place of delivery** |  |  |  |  |  |  |
| Home | 20,053 (61.2) | 12,689 (38.8) | 17,778 (62.4) | 10,695 (37.6) | 2,275 (53.3) | 1,995 (46.7) |
| Health facility | 32,397 (63.5) | 18,585 (36.5) | 29,717 (64.2) | 16,557 (35.8) | 2,680 (56.9) | 2,028 (43.1) |
| Missing | 891 (59.8) | 600 (40.2) | 802 (59.9) | 538 (40.1) | 89 (58.7) | 62 (41.3) |
| **Caesarean-section delivery** |  |  |  |  |  |  |
| No | 50,845 (62.4) | 30,586 (37.6) | 45,951 (63.3) | 26,592 (36.7) | 4,894 (55.1) | 3,994 (44.9) |
| Yes | 2,422 (66.2) | 1,238 (33.8) | 2,278 (66.4) | 1,153 (33.6) | 144 (62.9) | 85 (37.1) |
| Missing | 74 (59.7) | 50 (40.3) | 68 (60.8) | 44 (39.2) | 6 (50.0) | 6 (50.0) |
| **Postnatal care** |  |  |  |  |  |  |
| No | 37,029 (63.7) | 21,125 (36.3) | 33,347 (64.7) | 18,217 (35.3) | 3,682 (55.9) | 2,908 (44.1) |
| Yes | 16,240 (60.3) | 10,713 (39.7) | 14,883 (60.9) | 9,542 (39.1) | 1,357 (53.7) | 1,171 (46.3) |
| Missing | 71 (66.7) | 36 (33.3) | 67 (69.1) | 30 (30.9) | 4 (44.1) | 6 (55.9) |
| **Breastfeeding status** |  |  |  |  |  |  |
| Still breastfeeding | 32,438 (63.1) | 18,981 (36.9) | 31,371 (63.2) | 18,254 (36.8) | 1,067 (59.5) | 728 (40.5) |
| Not breastfeeding | 19,575 (62.0) | 11,999 (38.0) | 15,787 (64.2) | 8,792 (35.8) | 3,788 (54.1) | 3,208 (45.9) |
| Never breastfeeding | 1,233 (59.7) | 831 (40.3) | 1,059 (60.5) | 692 (39.5) | 173 (55.6) | 139 (44.4) |
| Missing | 95 (60.6) | 62 (39.4) | 80 (61.0) | 51 (39.0) | 15 (58.3) | 11 (41.7) |
| **Current contraceptive use** |  |  |  |  |  |  |
| Not using | 27,253 (57.7) | 19,975 (42.3) | 27,253 (57.7) | 19,975 (42.3) | -^[[4]](#footnote-4)^ | - |
| Using | 21,045 (72.9) | 7,814 (27.1) | 21,045 (72.9) | 7,814 (27.1) | - | - |
| Pregnant | 5,043 (55.2) | 4,085 (44.8) | - | - | - | - |
| **Time from last birth to date of interview (Mean±SD)** | 23.2±15.6 | 22.0±15.8 | 22.3±15.7 | 20.6±15.8 | 32.2±11.7 | 31.7±11.6 |

1. All women- women of reproductive age groups (15-49 years) who had at least two consecutive singleton children with the last child born five years preceding the survey. These women could be non-pregnant, pregnant, or postpartum women. [↑](#footnote-ref-1)
2. Non-pregnant women- women who had at least two consecutive singleton children with the last child born five years preceding the survey and were not pregnant during the time of interview. [↑](#footnote-ref-2)
3. Pregnant women- women who had at least two consecutive singleton children with the last child born 5 years preceding the survey and were pregnant during the time of interview. [↑](#footnote-ref-3)
4. Pregnant women are not eligible for current contraception use [↑](#footnote-ref-4)
