# Supplementary material for: Interbirth interval and maternal anaemia in 21 sub-Saharan African countries: A fractional-polynomial analysis
Source: PLoS One. 2022 Sep 23;17(9):e0275155. doi: 10.1371/journal.pone.0275155 (PMC9506648; doi:10.1371/journal.pone.0275155)
Supplement: S3 Table — (DOCX) [file pone.0275155.s004.docx]

S3 Table. The number of samples included and maternal anaemia level in each Sub-Saharan African Country 2010-2017.

| Country | Survey year | Sample size | Weighted prevalence of anaemia (%) | Short interbirth^[[1]](#footnote-1)^ interval (%) | Long interbirth^[[2]](#footnote-2)^ interval (%) |
| --- | --- | --- | --- | --- | --- |
| **Southern Africa** |  | **20,869** | **34.1** | **11.1** | **23.0** |
| Lesotho | 2014 | 790 | 27.5 | 12.5 | 35.0 |
| Malawi | 2010, 2015/16 | 6,766 | 29.0 | 11.8 | 18.1 |
| Mozambique | 2011 | 5,969 | 53.5 | 12.1 | 14.9 |
| Namibia | 2013 | 1,181 | 19.4 | 12.8 | 38.0 |
| South Africa | 2016 | 672 | 30.6 | 11.0 | 40.8 |
| Zimbabwe | 2010/11, 2015 | 6,162 | 24.5 | 8.8 | 31.9 |
| **Western Africa** |  | **19,898** | **49.7** | **14.4** | **13.5** |
| Benin | 2011 | 2,236 | 42.1 | 12.9 | 14.5 |
| Burkina Faso | 2010 | 4,224 | 51.0 |  |  |
| Ghana | 2014 | 1,577 | 43.3 | 11.0 | 25.8 |
| Guinea | 2012 | 1,988 | 54.2 | 10.6 | 15.2 |
| Cote d'Ivoire | 2011 | 1,898 | 55.3 | 13.6 | 19.3 |
| Mali | 2012 | 2,809 | 53.2 | 17.8 | 13.1 |
| Niger | 2012 | 3,064 | 45.4 | 21.0 | 5.9 |
| Senegal | 2010/11, 2017 | 4,949 | 52.4 | 16.2 | 13.3 |
| **Eastern Africa** |  | **29,903** | **29.3** | **16.9** | **16.1** |
| Ethiopia | 2011, 2016 | 12,039 | 23.8 | 16.7 | 15.6 |
| Rwanda | 2010, 2014/15 | 4,560 | 18.2 | 16.1 | 15.8 |
| Tanzania | 2010, 2015/16 | 9,431 | 41.9 | 15.2 | 19.0 |
| Uganda | 2011, 2016 | 3,873 | 29.0 | 22.8 | 1.5 |
| **Central Africa** |  | **11,024** | **39.9** | **20.0** | **10.8** |
| Burundi | 2016 | 3640 | 43.6 | 15.5 | 9.4 |
| Cameroon | 2011 | 2998 | 37.8 | 18.5 | 14.4 |
| DR. Congo^[[3]](#footnote-3)^ | 2013 | 4386 | 38.1 | 10.9 | 9.5 |
| **Total** | **2010-2016** | **85,215** | **36.9** | **15.2** | **16.5** |

1. Short interbirth interval is defined for having <24 months [↑](#footnote-ref-1)
2. Long interbirth interval refers for ≥60 months [↑](#footnote-ref-2)
3. Democratic Republic of Congo [↑](#footnote-ref-3)
